# Effectiveness of Surgical Drains in Obese Patients Undergoing Lumbar Discectomy

**DOI:** 10.7759/cureus.66572

**Published:** 2024-08-10

**Authors:** Aryadev Jayakrishnan, Hariprasad Seenappa, Manoj K Ramachandraiah

**Affiliations:** 1 Department of Orthopaedics, Sri Devaraj Urs Academy of Higher Education and Research, Kolar, IND

**Keywords:** bmi, odi, vas, lumbar discectomy, hematoma, drain, obesity

## Abstract

Background

The placement of postoperative drains after spine surgery is a contentious issue, and its application has changed over time. Obesity itself is an independent risk factor for postoperative complications. Hematomas in the surgical wound are a complication that may necessitate revision surgery. Orthopaedic surgeons frequently use closed drainage in orthopaedic surgery to prevent the formation of a hematoma. It remains unclear whether drains reduce postoperative complications and improve clinical outcomes, especially in obese patients who are already at risk of such complications.

Objectives

To assess the incidence of surgical site infections (SSI) after lumbar discectomy in obese and morbidly obese patients with or without postoperative wound drainage and compare functional outcomes between both groups.

Methodology

A hospital-based retrospective study was conducted among 84 patients with obesity who underwent single-level lumbar discectomy at R. L. Jalappa Hospital and Research Centre, Kolar, India from May 2022 to April 2023. Drains were used for patients in Group A and avoided for patients in Group B.

Results

Postoperative C-reactive protein (CRP) levels in the non-drainage group were much higher than in the drainage group and were statistically significant. There was a statistically significant association found between body mass index (BMI) and postoperative SSI. In Group A, only three patients had SSI while in Group B, eight patients suffered from SSI.

Conclusion

Closed suction drains were shown to have a positive impact in reducing SSI in patients with obesity. Drain tip culture may be beneficial in detecting SSI at the earliest. Hence, we believe that closed suction drainage can be considered as a standard protocol in obese patients.

## Introduction

The overall complication rate after spine surgery ranges between 7% and 20% [1]. Avoiding such complications is a major concern for any surgeon. Hematomas in surgical wounds are a complication with an incidence of 0.2-2.9% [2,3]. Though symptomatic in only 0.1-0.24% of cases [4], it can cause neurological sequelae such as bowel and bladder dysfunction, saddle anaesthesia, motor weakness, and wound healing issues like surgical site infection (SSI) and delayed healing [5,6].

On the other hand, obesity and morbid obesity alone are risk factors for complications following spine surgery both during intra-operative and postoperatively [7,8]. Longer duration of surgery and increased blood loss in obese patients [9,10] can lead to hematoma formation which has been identified as a risk factor for postoperative complications [11].

Closed suction drain has been utilized extensively in orthopaedic surgery, especially by spine surgeons, to prevent hematomas from forming [12-15]. However, their usage has been linked to drawbacks such as retrograde infection, higher postoperative blood loss [3], skin and deep tissue inflammation [4], extended length of stay and increased hospital expenditure [5]. Whether the use of drain improves clinical outcomes and lowers postoperative complications, particularly in obese patients who are already at risk of complication, remains to be seen.

In order to evaluate and compare the surgical outcome between drainage and non-drainage groups in obese patients, we retrospectively assessed the patients who had undergone single-level lumbar discectomy in terms of SSI rate, length of hospital stay, C-reactive protein (CRP) levels, Visual Analogue Scale (VAS) and Oswestry Disability Index (ODI).

## Materials and methods

Eighty-four patients considered obese and morbidly obese as per WHO definition [16] and who underwent elective single-level lumbar discectomy were retrospectively analysed after retrieving data from the medical records department with a minimum of three months of follow-up during the period May 2022-April 2023. The study was initiated after obtaining approval from the Institutional Ethics Committee at Sri Devaraj Urs Academy of Higher Education and Research (no. SDUMC/KLR/IEC/275/2023-2024). Inclusion criteria included patients above the age of 18 years who had undergone elective single-level lumbar discectomy and had the presence or absence of drain as documented in medical records. Patients suspected of spinal infection prior to the procedure (epidural abscess, discitis), patients who underwent instrumented spinal surgery, patients on long-term steroids and patients with an intra-operative dural breach leading to cerebrospinal fluid leak were all excluded from the study.

Patients were assessed upon the data retrieved from the picture archiving and communication system (PACS) and were divided into two groups. Group A included patients with a drain inserted. Two drain tubes, one in the subfascial layer and another in the subcutaneous layer, connected to a single collection unit were used as a standard practice in all patients in Group A. Group B included patients in whom the drain was not used. All patients who underwent single-level lumbar spine discectomy were operated on by the single same spine surgeon. CRP levels were assessed and compared for all patients in both groups on postoperative day 3. Closed suction drains were removed after an average of five days postoperatively or when the collection was less than 50mL/day for patients in Group A based on the operating surgeon's experience. Drain tips were sent for culture and sensitivity tests.

The primary outcome was the number of wound infections. The development of erythema, induration, pain, and culture-positive serous or contaminated discharge indicated wound infection. SSI was usually considered within 30 days of the surgery. Superficial SSI was usually identified with complaints of pain, local tenderness, redness and local rise of temperature at the surgical site involving skin and subcutaneous tissue. Deep SSI affected the fascia and muscle presenting with fever and abscess formation along with pain and tenderness. The diagnosis of SSI in patients included in the study was confirmed by drain tip culture growth and ultrasound (USG). Culture swab sticks were taken for patients who developed discharge from the surgical site. Patients of the two groups were compared based on rates of SSI, levels of CRP, and scores of VAS and ODI in postoperative and first postoperative follow-up phases at outpatient departments.

Data entry and analysis

Microsoft Excel (Microsoft® Corp., Redmond, WA) was used for data entry and Statistical Package for the Social Sciences (IBM SPSS Statistics for Windows, IBM Corp., Version 20.0, Armonk, NY)S was used for data analysis. All continuous variables were summarized using mean and standard deviation (SD). Categorical variables were summarized using proportions. Comparison of continuous variables across study groups (drain vs no-drain) was done using the Chi-square test. Comparison of categorical variables across study groups (drain vs no-drain) was done using the Chi-square test. A p-value of <0.05 was considered statistically significant.

## Results

Group A included 25 males and 17 females, while 23 were males and 19 were females in Group B. The mean age among Group A was 59.76 ± 10.8 years and among Group B was 62.24 ± 9.7 years. The mean hospital stay duration among Group A was 14.8 ± 2.7 and among Group B was 11.8 ± 4.1 days. Group A patients had a longer mean duration of hospital stay which was statistically significant.

Out of the total, in Group A 32 and 10 patients were obese and morbidly obese respectively based on body mass index (BMI) calculation while in Group B 30 and 12 patients were, respectively, obese and morbidly obese as indicated in Table 1.

**Table 1:** BMI-wise distribution among study participants (n=84)

BMI	Obese	Morbidly obese
Group A	32	10
Group B	30	12

**Figure 1:**
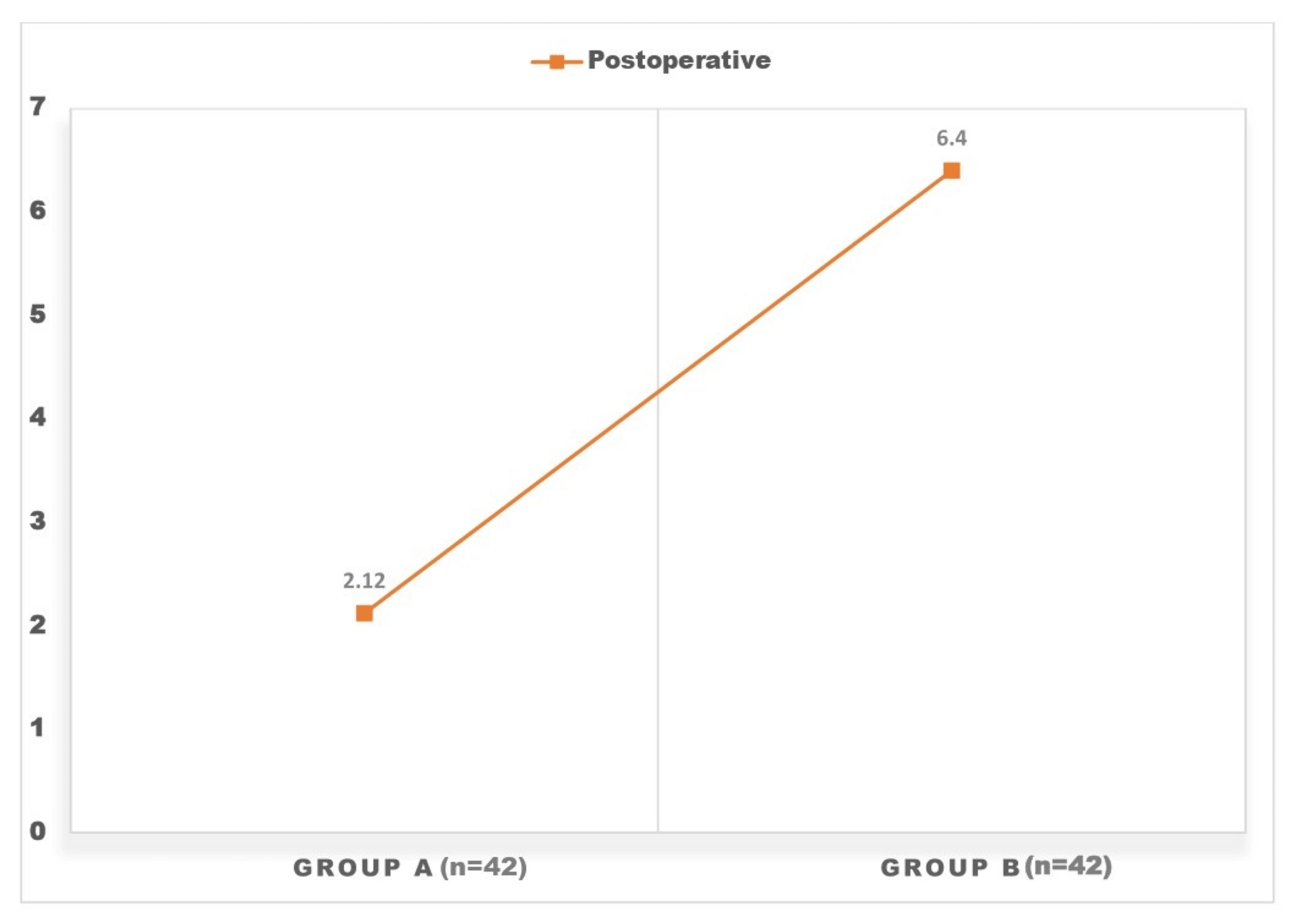
Graph showing the comparison of CRP levels between patients in Groups A and B during the postoperative period (p-value of 0.0001).

Results from the comparison of CRP levels between patients in Groups A and B as indicated in Figure 1 during the postoperative period showed a statistically significant difference between the drain and no drain group (p-value of 0.0001).

As shown in Table 2, the number of cases of postoperative infection was more in Group B which was found to be statistically significant.

**Table 2:** Comparison of postoperative SSI between Groups A and B (n=84) SSI: surgical site infection

Postoperative SSI	Group A	Group B	p-value
Yes	3	8	0.0107
No	39	34	

There was a statistically significant association found between BMI and postoperative infection as shown in Table 3. In Group A only three patients had SSI while in Group B eight patients suffered from SSI. In both groups specifically, morbidly obese patients were more prone to SSI.

**Table 3:** Comparison of postoperative SSI and BMI of patients in both groups (n=84) SSI: surgical site infection

Postoperative SSI	Obese	Morbidly obese	p-value
Group A	1	2	0.0465
Group B	3	5	

Among the 11 patients who had SSI, nine were superficial and two were deep (Table 4) as confirmed by USG. Both the patients who had deep SSI were morbidly obese and were in the no-drain group. The most common organism isolated was *Staphylococcus aureus* (n=9). *Streptococcus* species were isolated from two cases. All patients diagnosed with SSI were managed with drain tip culture-specific antibiotics. Two patients with deep SSI underwent wound re-exploration and debridement.

**Table 4:** Surgical site infections (SSIs) (n=11)

SSI	Group A	Group B
Superficial	3	6
Deep	0	2

Comparison of VAS score (n=84)

**Figure 2:**
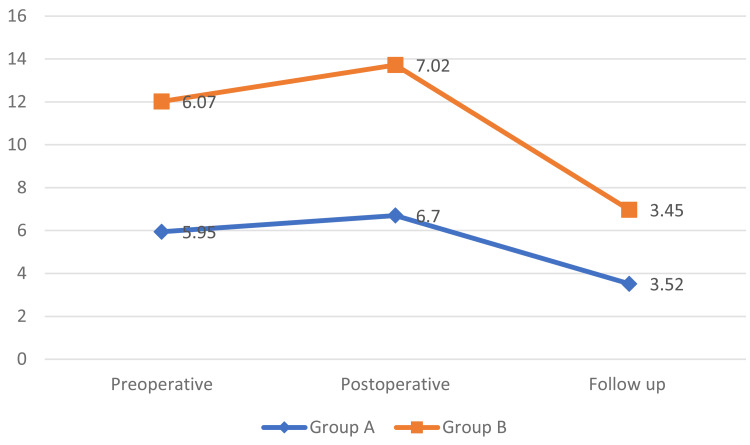
Graph showing the comparison of VAS scores between patients of Groups A and B during preoperative, postoperative and follow-up periods VAS: Visual Analogue Scale

No statistically significant difference was found in VAS scores between the groups during preoperative (p-value of 0.439), postoperative (p-value of 0.653) and follow-up (0.859) duration among both groups as noted in Figure 2.

Comparison of ODI scores (n=84)

**Figure 3:**
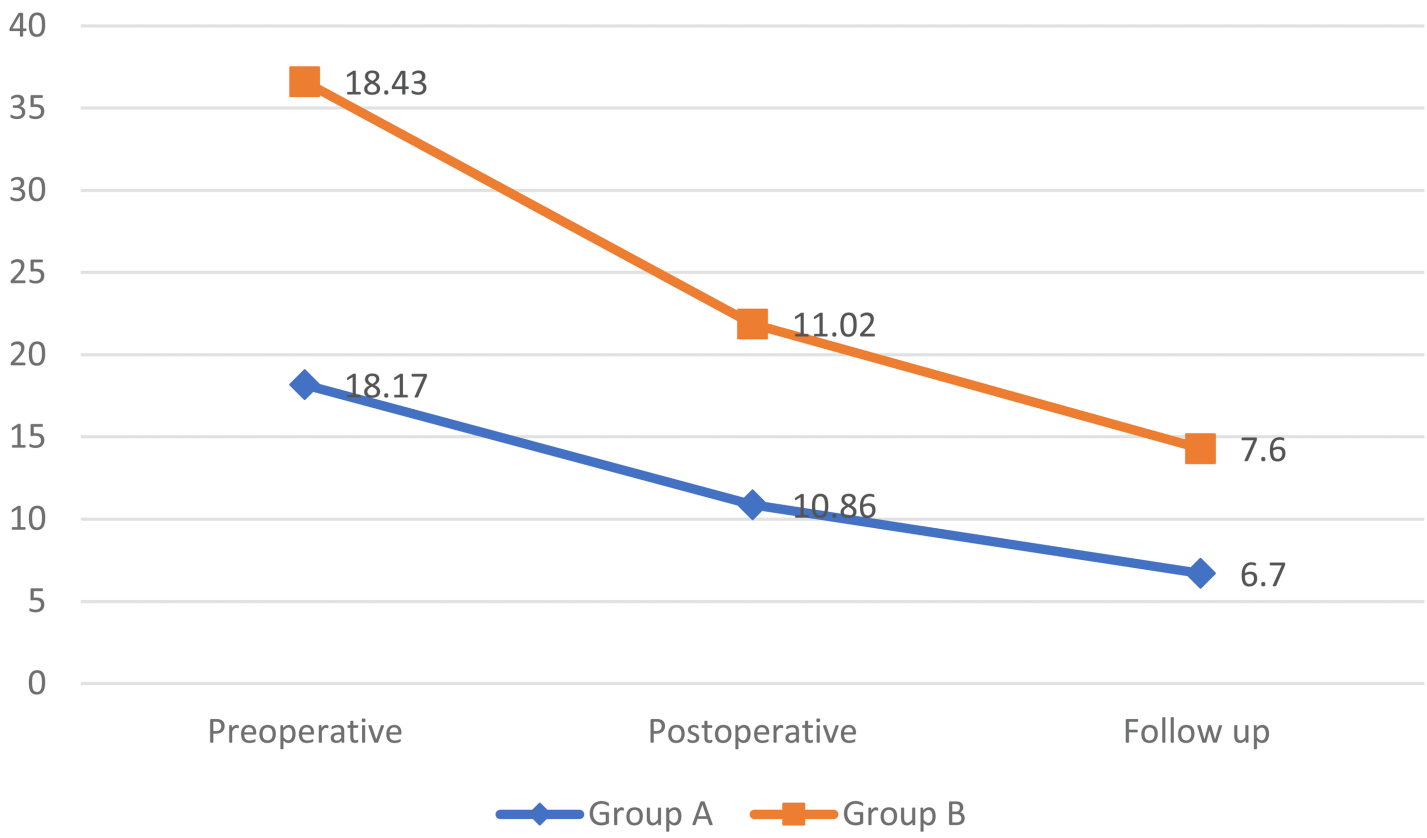
Graph showing the comparison of ODI scores between patients of Groups A and B during preoperative, postoperative and follow-up periods ODI: Oswestry Disability Index

Comparison of ODI scores between patients of Groups A and B during preoperative (p-value = 0.754), postoperative (p-value = 0.801) and follow-up period (p-value =0.230) showed no statistically significant difference between both the groups as shown in Figure 3.

## Discussion

Obesity is defined by WHO on the basis of an individual’s BMI. BMI between the range of 25 and 29.9 kg/m^2^ are categorized as overweight, between 30 and 40 kg/m^2^ as obese and those with more than 40 kg/m^2^ are morbidly obese. Spine surgery in obese patients has been related to increased duration of surgery, blood loss, treatment cost, rate of surgical SSI and higher risk of mortality [17]. There are probably many confounding factors that contribute to the complex association between obesity and higher incidence of postoperative complications including SSI. Diabetes mellitus and coronary artery disease are some of the comorbidities more common in obese patients undergoing spinal surgery and independent risk factors for adverse outcomes that may occur after surgery [9]. Several studies have linked the increased incidence of postoperative infection to the presence of obesity, particularly morbid obesity [17-19]. A meta-analysis by Jiang et al. reviewing 24 studies reported a significant association between obesity and SSI [10]. Mehta et al. in a study concluded that the distribution of adipose tissue has an important role in the occurrence of SSI following lumbar spine surgery. The study found a significant relation between skin-to-lamina distance and the depth of subcutaneous tissue in developing infections as measured on MRI in cervical and lumbar procedures [20].

On the other hand, the use of closed drainage in spine surgery remains controversial [13]. It has been primarily used to prevent the formation of hematoma and consequent SSI [14]. Postoperative hematoma in spine surgery can also lead to devastating neurological sequelae. Hence, placing a subfascial drain to prevent such complications is a common practice among spine surgeons [21]. Although drains have the benefit of decreasing haematoma formation, fever, and pain during the early postoperative period and preventing neurological deterioration, there may be some disadvantages. Few studies have shown that drains could serve as a point of entry for bacteria and subsequent infection [22,23]. According to a meta-analysis, drains in orthopaedic surgeries may do more harm than good and their only proven benefit is a decrease in the number of dressing changes [24]. Moreover, drains also increase the duration of hospital stay, postoperative blood loss and need for transfusions.

A survey conducted by Diab et al., among spine surgeons to understand the common patterns of surgical drain use in spine deformity surgeries, concluded that the decision to use a drain was made based on the surgeon’s habits developed during their training [25]. Another analysis by von Eckardstein et al. to assess the factors influencing a spine surgeon whether to use or not a drain concluded that the degree of haemostasis, surgical procedure and size of the wound were the most influential factors [21].

A prospective randomized trial of 50 patients reported by Mirzai et al. suggested that using a closed drain brought down hematoma formation detected using MRI on the first day after surgery. The study showed 89% of patients without a drain had hematoma and only 36% of patients with a drain developed hematoma formation. This indicated that the rate of absence of hematoma increased from 11% to 64% after using a drain [26].

We studied and compared the rate of SSI in obese and morbidly obese patients who underwent single-level lumbar discectomy with and without the usage of closed suction drainage. In our study, all patients who underwent single-level lumbar discectomy were either obese (n=62) or morbidly obese (n=22). To the best of our knowledge, the correlation between the usage of the drain in obese and morbidly obese patients following lumbar discectomy and SSI has never been studied.

Payne et al. in 1996 randomized 200 patients who underwent lumbar laminectomy based on whether a drain was used or not and found that among 103 patients with a drain, two patients (1.9%) developed wound infections and 1 of 97 patients (1.0%) without a drain had wound infection [27]. In 2010, a retrospective analysis by Kanayama et al. of 560 patients who had undergone lumbar spine decompression or discectomy showed that among the 298 patients with drains and 262 patients without drains, none of the patients developed wound infection [13]. Both authors came to the conclusion that closed suction drains provided no benefit in relation to rates of infection.

In our study, the overall incidence of SSI was 13.09% which was statistically significant (p=0.016). In the drainage group, the incidence was 7.14% (n=3) and in the non-drainage group, the incidence was 19.04% (n=8) which was found to be statistically significant. Therefore, we believe that the use of closed suction drainage in obese patients undergoing single-level lumbar discectomy may be beneficial in preventing SSI.

Kumar et al. assessed parameters like haemoglobin drop, CRP levels, and VAS for pain between the drainage and non-drainage groups in lumbar trauma surgery and found no significant difference among them. Furthermore, no difference was found between the groups in the duration of admission, rate of SSI, and risk of developing clinically significant haematoma or neurological deterioration [28]. Choi et al. conducted a study on whether surgical drain is useful following lumbar surgery and analysed parameters such as levels of CRP, rates of infection, preoperative and postoperative VAS scores and duration of hospital stay after operation and found no statistically significant difference among both the groups. However, he concluded that the use of closed drains didn’t elevate postoperative infection and drain tip cultures allowed to start appropriate antibiotics at an early stage [29]. In our study, we found no significant difference either in ODI or in VAS scores in the postoperative period and at three months follow-up in both groups.

Adogwa et al. in their retrospective study on 139 adult patients with spinal deformities who underwent spine surgery compared operative variables like postoperative length of stay between drain and no-drain groups. He concluded that the number of days spent in the hospital was significantly higher in the drain group [30]. Payne et al. in their study concluded that the duration of hospital stay was significantly longer in patients for whom a closed suction drain was used after undergoing single-level lumbar discectomy [27]. In our study, patients with closed drainage had a longer duration of hospital stay which was statistically significant.

Though our study demonstrated significant benefits of closed drainage in obese patients in preventing SSI, it is not without limitations. Firstly, our study is a retrospective analysis and is subjected to the weaknesses of retrospective reviews. Large prospective randomized control trials with longer follow-ups are required to draw definitive conclusions. The sample size in our study is small scale thereby limiting our ability to make firm conclusions. Data pooled from multiple centres may increase the number of patients and overcome this limitation. Patient comorbidities like diabetes which could have influenced the infection rate were not considered which could have influenced the results.

## Conclusions

In conclusion, the use of closed suction drainage after single-level lumbar discectomy in obese and morbidly obese patients who are already at risk of SSI can have a beneficial effect in reducing infection by preventing hematoma formation. Drain tip culture may also be beneficial in detecting SSI at the earliest. We did not find any difference in functional outcomes in terms of VAS and ODI scores in both groups. Hence, we believe that closed suction drainage can be considered as a standard protocol in obese patients undergoing single-level lumbar discectomy.
